# Primer containing dimethylaminododecyl methacrylate kills bacteria impregnated in human dentin blocks

**DOI:** 10.1038/ijos.2016.43

**Published:** 2016-11-04

**Authors:** Chen Chen, Lei Cheng, Michael D Weir, Nancy J Lin, Sheng Lin-Gibson, Xue-Dong Zhou, Hockin HK Xu

**Affiliations:** 1 State Key Laboratory of Oral Diseases, West China School of Stomatology, Sichuan University, Chengdu, China; 2 Biomaterials & Tissue Engineering Division, Department of Endodontics, Periodontics and Prosthodontics, University of Maryland Dental School, Baltimore, USA; 3 Biomaterials Group, Biosystems and Biomaterials Division, National Institute of Standards & Technology, Gaithersburg, USA; 4 Center for Stem Cell Biology & Regenerative Medicine, University of Maryland School of Medicine, Baltimore, USA; 5 Department of Mechanical Engineering, University of Maryland, Baltimore County, USA

**Keywords:** antibacterial bonding agent, dental restoration, dentin bond strength, dimethylaminododecyl methacrylate, killing bacteria in dentin, *Streptococcus mutans*

## Abstract

Antibacterial dimethylaminododecyl methacrylate (DMADDM) was recently synthesized. The objectives of this study were to: (1) investigate antibacterial activity of DMADDM-containing primer on *Streptococcus mutans* impregnated into dentin blocks for the first time, and (2) compare the antibacterial efficacy of DMADDM with a previous quaternary ammonium dimethacrylate (QADM). Scotchbond Multi-Purpose (SBMP) bonding agent was used. DMADDM and QADM were mixed into SBMP primer. Six primers were tested: SBMP control primer P, P+2.5% DMADDM, P+5% DMADDM, P+7.5% DMADDM, P+10% DMADDM, and P+10% QADM. *S. mutans* were impregnated into human dentin blocks, and each primer was applied to dentin to test its ability to kill bacteria in dentinal tubules. Bacteria in dentin were collected *via* a sonication method, and the colony-forming units (CFU) and inhibition zones were measured. The bacterial inhibition zone of P+10% DMADDM was 10 times that of control primer (*P*<0.05). CFU in dentin with P+10% DMADDM was reduced by three orders of magnitude, compared with control. DMADDM had a much stronger antibacterial effect than QADM, and antibacterial efficacy increased with increasing DMADDM concentration. Dentin shear bond strengths were similar among all groups (*P*>0.1). In conclusion, antibacterial DMADDM-containing primer was validated to kill bacteria inside dentin blocks, possessing a much stronger antibacterial potency than the previous QADM. DMADDM-containing bonding agent was effective in eradicating bacteria in dentin, and its efficacy was directly proportional to DMADDM mass fraction. Therefore, DMADDM may be promising for use in bonding agents as well as in other restorative and preventive materials to inhibit bacteria.

## Introduction

Dental caries is the destruction of dental hard tissues caused by acidic products from bacterial fermentation of dietary carbohydrates.^1, 2^ Approximately 200 million tooth cavity restorations were performed annually in the United States, costing about $40 billion per year.^3^ Resin composites are popular tooth cavity filling materials due to their esthetics and direct-filling capabilities. The chemical, physical, and mechanical properties of composites have been improved significantly.^4, 5, 6, 7, 8, 9, 10, 11^ Nonetheless, about half of all restorations fail in less than 10 years, with secondary caries as one of the primary reasons.^12, 13, 14, 15^ Therefore, it would be highly desirable to improve the materials to reduce secondary caries and restoration failure.^11, 12, 13, 14, 15^

The concept of completely removing carious dentin during cavity preparation is no longer recommended.^13, 14^ Residual bacteria remaining in dentinal tubules of the relatively healthy part of the dentin make it impossible to achieve caries-free cavities through the complete caries removal approach.^13, 16^ Furthermore, minimally invasive techniques propose the least invasive surgical approaches to remove carious lesion, in order to preserve more tooth structure and protect the pulpal vitality.^14, 17, 18^ Several methods were developed to precisely remove caries-infected tissue while achieving maximum preservation of caries-affected tissues.^14^ However, while preserving more tooth structure is meritorious, it will also leave more residual bacteria in the prepared cavity.^19^ Therefore, it would be beneficial to develop antibacterial primers that can be applied into the tooth cavity to eradicate or significantly reduce bacterial load in the dentin, thereby minimizing the adverse effects of residual bacteria.

Adhesives are used to bond composite restorations to tooth structures. Previous studies have improved the adhesive compositions, bond strength, and the durability of the bonded interface.^20, 21, 22, 23, 24, 25, 26^ In order to impart an antibacterial property to inhibit biofilm growth, quaternary ammonium methacrylates (QAMs) were synthesized and incorporated into dental resins.^27, 28, 29, 30, 31^ Novel bonding agents containing 12-methacryloyloxydodecyl-pyridinium bromide (MDPB) were developed and showed a strong antibacterial activity.^27, 32^ Other investigators synthesized methacryloxyl ethyl cetyl dimethyl ammonium chloride (DMAE-CB) for incorporation into resins.^33^ Besides bonding agents, dental composites containing nanoparticles of quaternary ammonium polyethylenimine were also developed with antibacterial functions.^34^ Recently, a quaternary ammonium dimethacrylate (QADM) was synthesized and incorporated into resins, showing effective inhibition of dental plaque microcosm biofilm growth.^35^

With the purpose of killing residual bacteria inside dentinal tubules in tooth cavities, a previous study showed that a primer containing MDPB killed bacteria that had been impregnated into a dentin block.^16^ Recently, another primer containing QADM was also demonstrated to kill *Streptococcus mutans* impregnated into human dentin blocks.^36^ Therefore, such antibacterial bonding agents are promising to eradicate bacteria in tooth cavities in order to inhibit caries and protect the pulp, and they could be especially beneficial when using minimally invasive procedures. More recently, a new quaternary ammonium monomer dimethylaminododecyl methacrylate (DMADDM) was synthesized and shown to possess a highly potent antibacterial activity.^37^ However, the effect of a DMADDM-containing primer on the killing of bacteria impregnated into dentin blocks has not been reported.

The objectives of this study were to investigate the effects of a DMADDM-containing primer on the killing efficacy of *S. mutans* impregnated into dentin in comparison with the previous QADM. The following hypotheses were tested: (1) both DMADDM-containing primer and QADM-containing primer would be effective in killing *S. mutan* inside dentin, but DMADDM-containing primer would have a higher efficacy than QADM-containing primer in killing bacteria inside dentin; (2) increasing the DMADDM mass fraction in primer would increase the efficacy of killing bacteria inside dentin; (3) the killing of bacteria inside a tooth cavity could be achieved without compromising the dentin shear bond strength, compared with control primer without DMADDM.

## Materials and methods

### Synthesis of DMADDM

DMADDM was synthesized using a modified Menschutkin reaction method, in which a tertiary amine group was reacted with an organo-halide.^29, 35, 37^ Commercial 1-(dimethylamino)docecane (DMAD; Tokyo Chemical Industry, Tokyo, Japan) and 2-bromoethyl methacrylate (BEMA; Monomer-Polymer and Dajac Labs, Trevose, PA, USA) were used as the tertiary amine and the organo-halide, respectively. First, 10 mmol of DMAD and 10 mmol of BEMA were mixed in a 20 mL scintillation vial with a magnetic stir bar. The reaction mixture was stirred at 70 °C for 24 h.^37^ After the reaction was completed, the ethanol solvent was removed *via* evaporation. This process yielded DMADDM as a clear, colorless, and viscous liquid. Fourier transform infrared spectroscopy confirmed the reaction and the products in a previous study.^37^

### DMADDM incorporation into primer

Scotchbond Multi-Purpose (3M, St. Paul, MN, USA) was used as the parent bonding system (referred to as SBMP). According to the manufacturer, SBMP etchant contained 37% (all by mass) phosphoric acid. SBMP primer contained 35%–45% of 2-hydroxyethylmethacrylate (HEMA), 10%–20% of a copolymer of acrylic and itaconic acids, and 40%–50% of water. SBMP adhesive contained 60%–70% of bisphenol A diglycidyl methacrylate (BisGMA), 30%–40% of HEMA, tertiary amines and photo-initiator. The present study tested the killing of bacteria *via* primer, hence DMADDM was incorporated into the primer. The SBMP etchant and adhesive were unmodified. The following experimental bonding systems, which contained SBMP primer mixed with different mass fractions of DMADDM, were tested and compared with the control group:

1. SBMP primer (P; control).
2. P+2.5% (by mass) DMADDM.
3. P+5% DMADDM.
4. P+7.5% DMADDM.
5. P+10% DMADDM.
6. P+10% QADM.

QADM had been developed and investigated in previous studies^29, 36^ and served as a comparative control here, to determine the relative antibacterial potency of DMADDM, compared with the previous QADM. These mass fractions were selected following a previous study.^38^

### Dentin shear bond strength testing

The use of extracted human teeth was approved by the University of Maryland Institutional Review Board. Caries-free molars were cleaned and stored in 0.01% thymol solution. Flat mid-coronal dentin surfaces were prepared by cutting off the tips of crowns with a diamond saw (Isomet, Buehler, Lake Bluff, IL, USA).^35, 39^ Each tooth was embedded in a poly-carbonate holder (Bosworth, Skokie, IL, USA). The occlusal surface was ground on 320 grit silicon carbide paper until the occlusal enamel was removed. The dentin surface was etched with the SBMP echant for 15 s and then washed with water. A primer was applied and rubbed in for 15 s with a brush-tipped applicator. The solvent was removed with a stream of air for 5 s. Then the adhesive was applied and light-cured for 10 s (Optilux VCL 401; Demetron Kerr, Danbury, CT, USA). A 1.5 mm-thick stainless-steel iris with a central opening of 4 mm in diameter was placed on the adhesive-treated dentin surface.^40^ The central opening was filled with a composite (TPH; Caulk/Dentsply, Milford, DE, USA) and light-cured for 60 s.^36, 37^ The bonded specimens were stored in water at 37 °C for 1 day and then used for testing. A chisel connected to a Universal Testing Machine (MTS, Eden Prairie, MN, USA) was aligned to be parallel to the composite–dentin interface. Then a load increasing at a constant rate of 0.5 mm·min^−1^ was applied until the dentin surface and composite were separated. Dentin shear bond strength *S* was calculated as


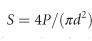


where *P* is the load for fracturing the bond, and *d* is the diameter of the composite.^36, 37, 40^

### Agar disk diffusion test

*S. mutans* is a cariogenic bacterium and is the primary causative agent of caries. The use of *S. mutans* (ATCC 700610; American Type Culture, Manassas, VA, USA) was approved by the University of Maryland Institutional Review Board. Agar disk diffusion test (ADT) was used to examine the antibacterial effect of uncured primers. Samples were prepared by adding 15 μL of stock bacteria to 15 mL of growth medium consisting of brain heart infusion (BHI) broth (Becton Dickenson, Franklin Lakes, NJ, USA) supplemented with 0.2% sucrose. After 24 h incubation at 37 °C with 5% (by volume) CO_2_, 0.4 mL of bacteria suspension was swabbed across a BHI agar plate (4 mm height and 90 mm diameter). A primer-impregnated paper disk was prepared by dropping 20 μL of a primer into a sterile paper disk with 1.5 mm thickness and 6 mm diameter, following previous studies.^16, 37^ The primer-impregnated paper disks were placed on agar plates with bacteria and incubated in 5% CO_2_ at 37 °C for 48 h. Bacteria inhibition zone size=(outer diameter of inhibition zone−paper disk diameter)/2, following previous studies.^16, 37^

### Bacteria impregnation into dentin blocks and scanning electron microscopy examination

Dentin blocks of ~4 mm × 4 mm × 0.5 mm were cut from the crown of extracted molars. Dentin blocks were cut at a distance of ~1 mm away from the pulp, with one surface facing the pulp, and the other surface facing the occlusal enamel. The dentin block was ground on a 1 500 grit silicon carbide abrasive paper to reduce the thickness to ~200 μm.^16, 36^ The dentin surface was treated with 37% phosphoric acid for 3 min and then thoroughly rinsed with distilled water for 60 s to remove the smear layer.^41^ The dentin samples were then sterilized with ethylene oxide (Anprolene AN 74i; Andersen, Haw River, NC, USA).

To simulate bacterial colonization in carious dentin, a 2 μL aliquot of *S. mutans* suspension, having a concentration of 10^10^ CFU per mL in BHI, was placed on the surface of the dentin block for 10 min for infiltration, following previous studies.^16, 36^ To prepare specimens for scanning electron microscopy (SEM) examination, first, the dentin blocks were immersed in 1% glutaraldehydein phosphate-buffered saline (PBS) at 4 °C for 4 h. Then, graded ethanol dehydrations were applied to the dentin. Finally, the dentin blocks were rinsed with 100% hexamethyldisilazane twice, and sputter-coated with gold (Quanta 200; FEI, Hillsboro, OR, USA). Dentin blocks with and without bacteria impregnation were both examined.^36^

### Bacteria viability staining assay

Two microliters of *S. mutans* suspension at 10^10^ CFU per mL was placed on a dentin block to infiltrate into dentin, as described above. Then a primer was applied to the dentin and left for 20 s. Two microliters of primer was used for each dentin block. Six dentin blocks were used to test each primer (*n*=6). The dentin blocks were then stained using a live/dead bacterial viability kit (Molecular Probes, Eugene, OR, USA) following the manufacturer's protocol. Live bacteria were stained with Syto 9 to show a green fluorescence. Bacteria with compromised membranes were stained with propidium iodide to show a red fluorescence. The stained samples were imaged *via* an epifluorescence microscope (Eclipse TE2000-S; Nikon, Melville, NY, USA).^37, 42^

### Method to harvest bacteria from inside dentin blocks

A 2 μL *S. mutans* suspension at 10^10^ CFU per mL in BHI was impregnated into a dentin block as described above.^16, 36^ Each impregated dentin block was placed into a tube with 2 mL of cysteine peptone water (CPW). Then the bacteria were harvested by sonication (3510R-MTH; Branson Ultrasonics, Danbury, CT, USA) at a frequency of 40 kHz for 5 min and vortexing at the maximum speed for 20 s using a vortex mixer (Fisher, Pittsburgh, PA, USA), following a previous study.^36^ The harvested bacteria were serially diluted in CPW and plated on BHI agar plates.^36^ The agar plates were incubated at 37 °C with 5% CO_2_ for 3 days, and the number of colonies was counted on the plates. This yielded the CFU counts harvested from the dentin block by the sonication method.^36^

To examine whether the sonication method harvested all the bacteria inside the dentin, the dentin after sonication was then cut into small pieces and homogenized in 500 μL of CPW, following previous studies.^16, 36^ The cutting and homogenizing method was previously shown to harvest the bacteria in dentin blocks.^16, 36^ The solution with small dentin pieces was vortexed using the vortex mixer at maximum speed for 20 s.^16, 36^ The purpose was to harvest any residual bacteria that remained in the dentin after the sonication method. The homogenized solution was plated on agar plates and incubated for 3 days, and the colonies were counted. If the residual CFU was negligible compared with the CFU from the sonication method, it would indicate that the sonication method could effectively harvest the bacteria from the blocks. The rationale for this experiment was that the sonication method for harvesting the bacteria in dentin is a simpler method. If the sonication method could effectively harvest bacteria from the dentin, then the steps of cutting and homogenizing the dentin block could be avoided.

### Effects of primers on the killing of *S. mutans* impregnated in dentin

As described above, 2 μL of *S. mutans* at 10^10^ CFU per mL was placed on a dentin block to impregnate the bacteria into dentinal tubules. Then a primer was applied to the dentin. The dentins were used for CFU measurement using the sonication method.^36^ Dentin blocks with *S. mutans* impregnation and primer application were placed into tubes with 2 mL of CPW, and the bacteria were harvested by the sonication method. Then, the bacterial suspensions were serially diluted in CPW and plated on BHI agar plates. After incubating the agar plates at 37 °C with 5% CO_2_ for 3 days, the number of colonies were counted, as in a previous study.^36^ Six dentin blocks were tested for each primer (*n*=6).

### Statistical analysis

One-way and two-way analyses of variance were performed to detect the significant effects of the variables. The Kolmogoroff–Smirnoff test was used to examine the normal distribution of data. Tukey's multiple comparison was used to compare the data at *P* value of 0.05. Standard deviations (SD) served as the estimate for measurement uncertainties for each method.

## Results

**Figure 1:**
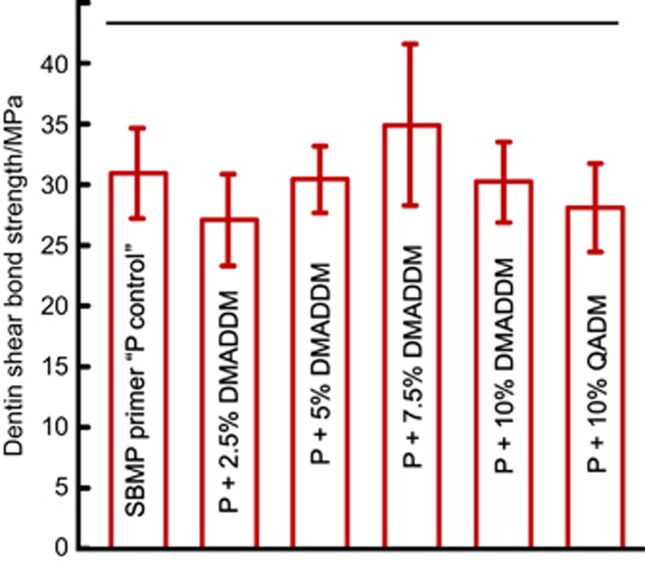
**Dentin shear bond strength.** Mean±SD; *n*=10. Horizontal line indicates no significant differences (*P*>0.1). Therefore, adding DMADDM and QADM into primer did not compromise the dentin bond strength. DMADDM, dimethylaminododecyl methacrylate; QADM, quaternary ammonium dimethacrylate; SD, standard deviations.

Dentin shear bond strengths using the six primers are shown in Figure 1 (mean±SD; *n*=10). The same SBMP adhesive was used for all groups, and the only difference was the primer. All six groups were assessed *via* the Kolmogoroff–Smirnoff test and all groups presented normal distribution in the data. All six groups were not significantly different from each other (*P*=0.389). The results showed that adding DMADDM and QADM into the primer did not adversely affect the dentin bond strength (*P*>0.1).

**Figure 2:**
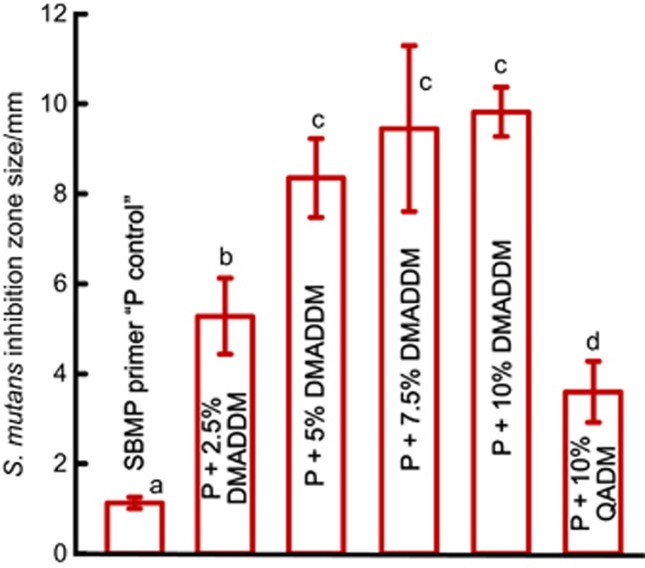
**Antibacterial activity of uncured primers in agar disk diffusion test.** The control group showed a small inhibition zone. The QADM group had a wider inhibition zone. DMADDM yielded much wider inhibition zones for primers as the DMADDM concentration was increased. Each value is mean±SD (*n*=6). Values with dissimilar letters (a–d) are significantly different from each other (*P*<0.05). DMADDM, dimethylaminododecyl methacrylate; QADM, quaternary ammonium dimethacrylate; SD, standard deviations.

The antibacterial effects of the six primers were measured *via* ADT and the results are plotted in Figure 2 (mean±SD; *n*=6). Control primer had minimal inhibition zones. The antibacterial primers containing QADM or DMADDM had significantly larger inhibition zones (*P*<0.05). Primers containing 7.5% or 10% DMADDM had inhibition zone sizes nearly an order of magnitude greater than that of control primer (*P*<0.05).

**Figure 3:**
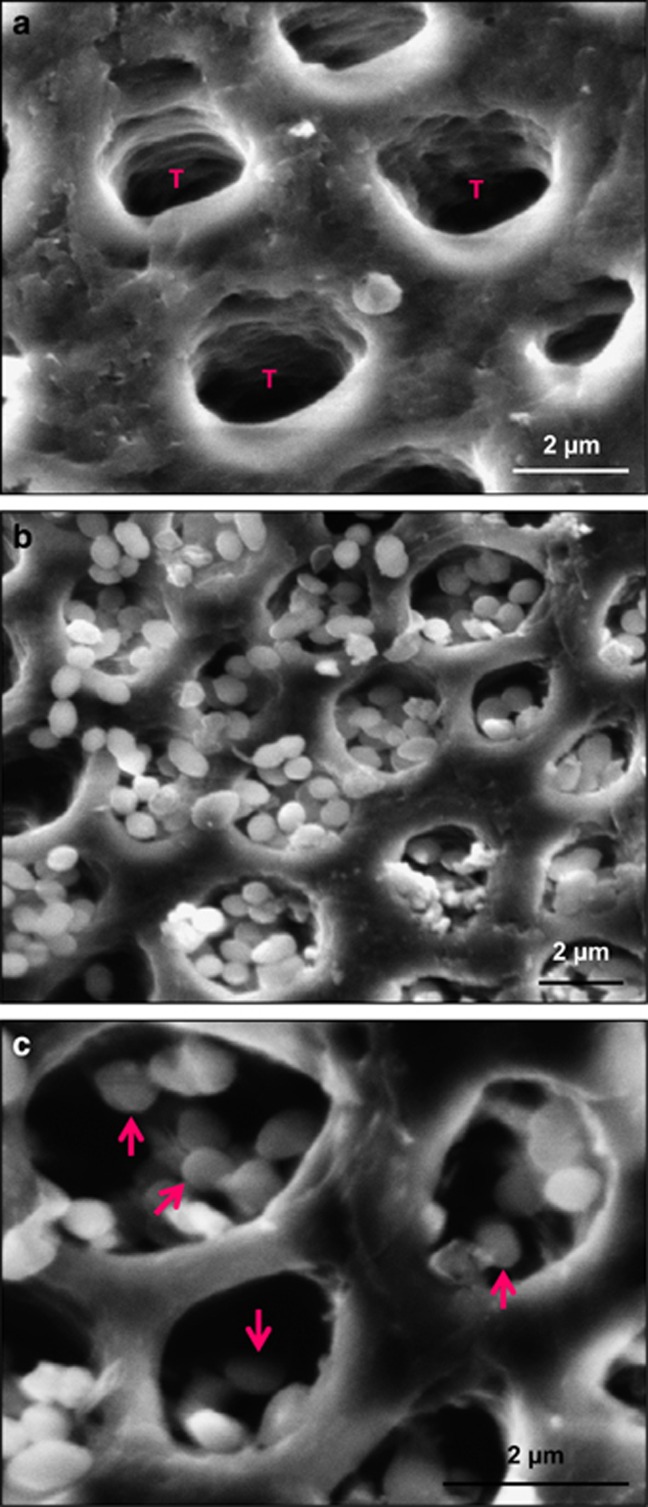
**Representative SEM images of dentin and** ***S. mutans*** **impregnation**. (**a**) Dentinal tubules before *S. mutans* impregnation, and (**b**) and (**c**) *S. mutans* were impregnated into dentinal tubules, at increasing magnification. T: dentinal tubules. Arrows indicate *S. mutans* in tubules. SEM, scanning electron microscopy.

Representative SEM micrographs of dentin blocks and *S. mutans* impregnation are shown in Figure 3: (a) examples of dentinal tubules “T” before *S. mutans* impregnation, (b) dentin with *S. mutans* impregnated into dentinal tubules, and (c) higher magnification showing *S. mutans* inside dentinal tubules. These images demonstrated that *S. mutans* were impregnated into the tubules.

**Figure 4:**
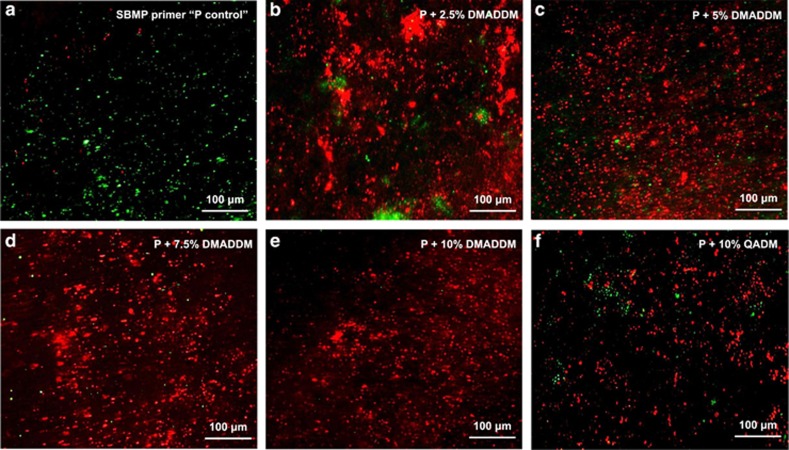
**Live/dead** ***S. mutans*** **staining images of dentin blocks treated with the six primers.** (**a**) SBMP primer ‘P' (control), (**b**) P+2.5% (by mass) DMADDM, (**c**) P+5% DMADDM, (**d**) P+7.5% DMADDM, (**e**) P+10% DMADDM, (**f**) P+10% QADM. Live bacteria were stained green, and compromised bacteria were stained red. Dentin with SBMP control primer had primarily live bacteria. In contrast, all the four groups containing DMADDM had mostly red bacteria, indicating that DMADDM-containing primer had a potent antibacterial activity to kill residual bacteria in dentin in the prepared tooth cavity. DMADDM, dimethylaminododecyl methacrylate; QADM, quaternary ammonium dimethacrylate.

Typical live/dead staining images of *S. mutans*-impregnated dentin after applying the six primers are shown in Figure 4a–4f. Live bacteria were stained green, and compromised bacteria were stained red. Dentin with SBMP control primer had much more live *S. mutans* than the other groups. Primer with QADM resulted in a remarkable increase in red staining of compromised *S. mutans*. Primers containing DMADDM had the most red staining, indicating that primers containing DMADDM likely killed the bacteria, and that DMADDM had a stronger antibacterial effect than QADM.

**Figure 5:**
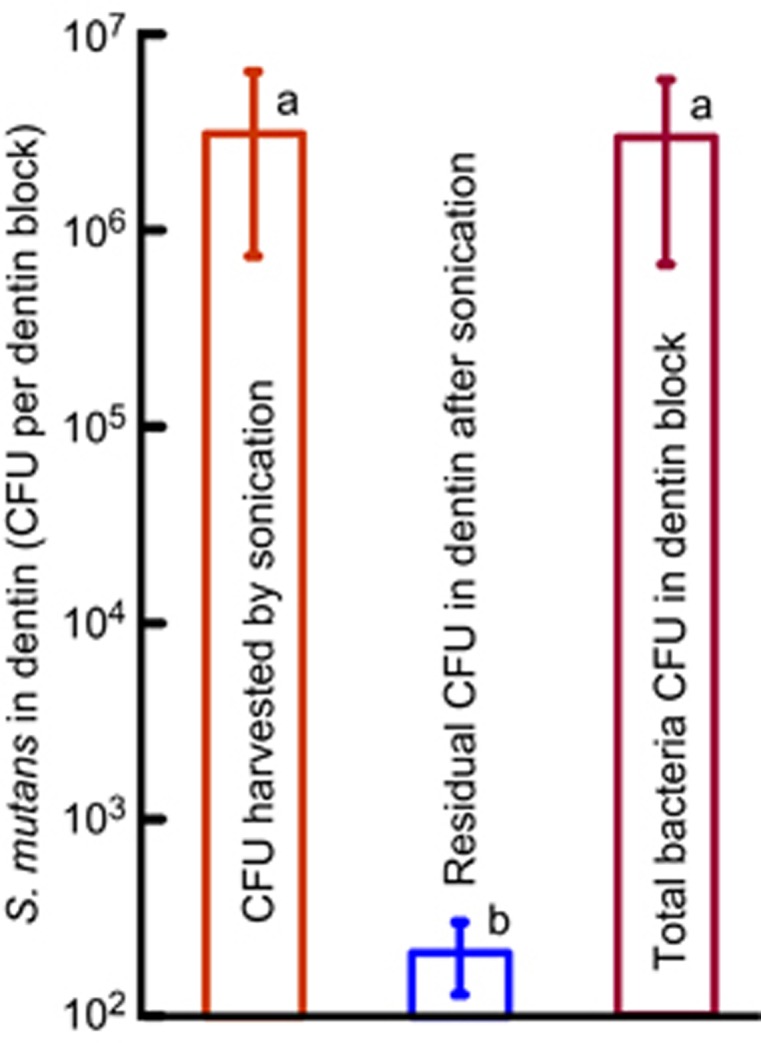
**Harvesting** ***S. mutans*** **that had been impregnated in dentin.** The sonication method harvested nearly all the *S. mutans* in dentin (mean±SD; *n*=6). The total CFU in dentin=the CFU harvested by sonication+the residual CFU in dentin. The residual bacteria in dentin after sonication were harvested by cutting the dentin into pieces and homogenization. CFU harvested by sonication was 99.99% of the total CFU. Therefore, the simple sonication method could be used to harvest the bacteria in dentin blocks. Values with dissimilar letters (a, b) are significantly different from each other (*P*<0.05). CFU, colony-forming units; SD, standard deviations.

The results of methods to harvest bacteria from dentin blocks impregnated with *S. mutans* are plotted in Figure 5 (mean±SD; *n*=6). The sonication method harvested nearly all the *S. mutans* in the dentin blocks. After sonication, the residual bacteria in dentin harvested by cutting the dentin into pieces and homogenization was four orders of magnitude lower than that harvested by sonication method. The total CFU in the dentin blocks were estimated as the CFU harvested by sonication plus the residual bacteria harvested by cutting the dentin into small pieces and homogenization. Hence, the CFU harvested by sonication was equal to ~99.99% of the total CFU in the dentin blocks. These results demonstrated that sonication was an effective bacterial harvesting method.

**Figure 6:**
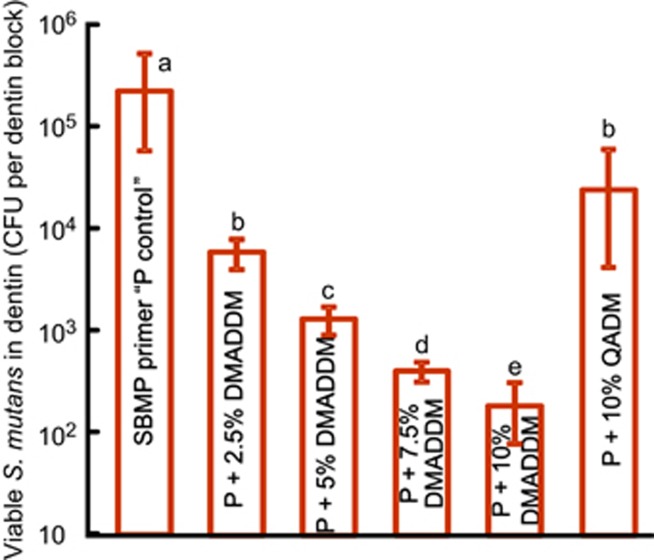
**Effects of different primers on** ***S. mutans*** **CFU in dentin blocks harvested by sonication** (mean±SD; *n*=6). DMADDM-containing primer effectively killed the bacteria impregnated in dentin blocks, reducing the culturable bacteria CFU harvested from dentin by three orders of magnitude compared with commercial control, and by two orders of magnitude compared with a previous QADM. Values with dissimilar letters (a–e) are significantly different from each other (*P*<0.05). CFU, colony-forming units; DMADDM, dimethylaminododecyl methacrylate; QADM, quaternary ammonium dimethacrylate; SD, standard deviations.

Figure 6 plots the CFU of *S. mutans* impregnated in dentin and measured using the sonication method for the six primer groups (mean±SD; *n*=6). Dentin with SBMP primer control had the highest CFU. CFU values from dentin treated with primers containing QADM were significantly lower than that for control (*P*<0.05). Increasing the DMADDM mass fraction in the primer significantly decreased the bacteria CFU (*P*<0.05). CFU of viable bacteria inside dentin for the primer with 10% DMADDM was three orders of magnitude lower than that of the SBMP control primer. At the same 10% mass fraction, the CFU of viable bacteria inside dentin for DMADDM group was much lower than that for QADM group, showing that DMADDM had a much higher killing efficacy than QADM.

## Discussion

This study showed that antibacterial primer containing DMADDM was much more effective than QADM in killing bacteria impregnated into dentin blocks. It is known that caries is a dietary carbohydrate-modified bacterial infectious disease.^43^ Its key feature is a dietary carbohydrate-induced enrichment of the plaque microbiota with organisms that would produce acid.^43, 44^ *S. mutans* have a vital role in the process of primary and secondary caries.^43, 44^ Residual bacteria often exist in prepared tooth cavities, and new bacteria can invade the tooth-restoration margins to cause secondary caries, which is a main reason for restoration failure.^15, 45^ Since dental primer directly contacts dentinal tubules, adding antibacterial agent into primer could help inhibit the growth of residual and invading bacteria.^16^ Thus, primer could serve as a promising vehicle for delivering antimicrobial agents.^27, 36^

DMADDM is a recently synthesized quaternary ammonium monomer with an alkyl chain length of 12.^37^ The antibacterial mechanism of quaternary ammonium salts is that they can cause bacteria lysis by binding to cell membrane.^34^ When the negatively charged bacterial cell contacts the positive charge of quaternary amine N^+^, the electric balance is disturbed to cause cytoplasmic leakage.^46^ In previous studies, many novel agents (MDPB, DMAE-CB, chlorhexidine particles, silver nanoparticles, and so on) were developed with antibacterial functions.^29, 35, 36, 37, 47, 48, 49, 50^ QADM is a quaternary ammonium dimethacrylate with less negative impact than a monomethacrylate on mechanical properties when it is incorporated and co-polymerized in a resin. In addition, a dimethacrylate is expected to have minimal monomer leach-out due to reactive groups on both ends of the molecule, as compared with a monomethacylate.^29, 35^ The present study showed that DMADDM was even more strongly antibacterial than QADM. DMADDM increased the bacteria inhibition zone and killed bacteria in dentinal tubules, which indicated the capability of cavity cleansing and disinfection by killing residual bacterial in tooth cavities.

The reason that DMADDM had a stronger antibacterial effect may be related to its longer carbon chain length. The carbon chain of quaternary ammonium needs to be long enough to penetrate the cell membranes to kill bacteria.^51, 52^ DMADDM had a chain length of 12, while QADM had a chain length of 2. Previous studies also indicated that the antibacterial activity would be enhanced if the carbon chain length was increased. For example, Xie *et al.* showed that increasing the chain length of quaternary ammonium monomer in glass ionomers significantly increased the antibacterial potency.^53^ Cheng *et al.* showed that DMADDM with a chain length of 12 possessed a much stronger antibacterial function than another monomer with a chain length of 6.^37^ These results are consistent with the present study on killing bacteria inside dentinal tubules via antibacterial primers, in which DMADDM was much more potent than QADM. Whether the killing of bacteria inside dentinal tubules can be further enhanced if the chain length is increased to >12 requires a further study.

Residual bacteria usually exist inside dentinal tubules after tooth cavity preparation, which could lead to pulpal damage.^16^ When the seal of a restoration is maintained, the bacterial numbers will likely diminish considerably, and caries is not likely to progress. However, for deep lesions approximating pulpal tissues and where infected dentin remains, it would be advantageous if bacterial numbers can be further reduced before restoration placement. Two problems exist regarding the use of traditional cavity disinfectants. First, whether traditional cavity disinfectants would reduce the adhesive bond strength remains a concern.^54, 55^ Second, traditional cavity disinfectants can not completely eliminate the viable microorganisms in the tooth cavity, and the antibacterial effect is not maintained for a long time.^56^ In this regard, an antibacterial primer containing a quaternary ammonium monomer is advantageous. First, it directly contacts the tooth structure and flows into the dentinal tubules, and could kill residual bacteria in dentin. Second, after photo-polymerization, the quaternary ammonium monomer is co-polymerized and remains in place to provide a long-term antibacterial effect. The present study showed that the novel DMADDM-containing primer could kill the bacteria impregnated in dentin blocks, reducing the viable bacteria CFU harvested from dentin by three orders of magnitude, compared with a commercial primer. This was achieved without negatively affecting the dentin bond strength. Therefore, DMADDM may be promising for use in various primers and bonding agents, as well as in cements, sealants and other dental resins.

It should be noted that the surrounding dental tissues as well as food and saliva may affect the antibacterial function. Carious dentin is often sclerotic with a poor permeability due to occlusion by whitlockite crystals; how the antibacterial primer can enter and penetrate such tubules to kill residual bacteria remains to be investigated. In addition, for deep cavities, whether the antibacterial primer will reach the pulp and what effect it will have on pulpal tissues need to be examined. Furthermore, at the restoration margin where the antibacterial bonding agent could potentially inhibit the invading bacteria, saliva could compromise the antibacterial efficacy. Indeed, coating the antibacterial resin with saliva moderately reduced the antibacterial activity, although biofilm growth and acid production were still substantially reduced.^57^ Further studies are needed to investigate these and other clinically relevant issues regarding the *in vivo* efficacy and long-term durability of antibacterial bonding agents.

## Conclusions

The present study investigated the effect of DMADDM mass fraction in primer on the efficacy of killing bacteria inside dentin blocks. The effect of DMADDM was compared with QADM on the killing of *S. mutans* impregnated into dentin blocks. The hypotheses were proven that while both DMADDM-containing primer and QADM-containing primer killed *S. mutan* inside dentin, the DMADDM-containing primer was much more potent; that increasing the DMADDM content in primer increased the efficacy of killing bacteria inside dentin; and that the dentin bond strength matched that of control without DMADDM. The novel DMADDM-containing primer reduced the viable bacterial CFU in dentin by three orders of magnitude, compared with a commercial primer. DMADDM-containing primer reduced bacteria CFU in dentin by two orders of magnitude, compared with the previous QADM. Therefore, DMADDM is a promising antibacterial monomer for use in primers as well as other resins such as adhesives and cements.
